# The Diagnostic Performance of Large Language Models and Oral Medicine Consultants for Identifying Oral Lesions in Text-Based Clinical Scenarios: Prospective Comparative Study

**DOI:** 10.2196/70566

**Published:** 2025-04-24

**Authors:** Sarah AlFarabi Ali, Hebah AlDehlawi, Ahoud Jazzar, Heba Ashi, Nihal Esam Abuzinadah, Mohammad AlOtaibi, Abdulrahman Algarni, Hazzaa Alqahtani, Sara Akeel, Soulafa Almazrooa

**Affiliations:** ^1^Department of Oral Diagnostic Sciences, Faculty of Dentistry, King Abdulaziz University, AlSulaimaniya, Jeddah, 22254, Saudi Arabia, 966 555821526; 2 Department of Public Health, Faculty of Dentistry, King Abdulaziz University, Jeddah, Saudi Arabia; 3 Department of Computer Science, Faculty of Computing and Information Technology, King Abdulaziz University, Jeddah, Saudi Arabia; 4 Faculty of Dentistry, King Abdulaziz University, Jeddah, Saudi Arabia

**Keywords:** artificial intelligence, ChatGPT, Copilot, diagnosis, oral medicine, diagnostic performance, large language model, lesion, oral lesion

## Abstract

**Background:**

The use of artificial intelligence (AI), especially large language models (LLMs), is increasing in health care, including in dentistry. There has yet to be an assessment of the diagnostic performance of LLMs in oral medicine.

**Objective:**

We aimed to compare the effectiveness of ChatGPT (OpenAI) and Microsoft Copilot (integrated within the Microsoft 365 suite) with oral medicine consultants in formulating accurate differential and final diagnoses for oral lesions from written clinical scenarios.

**Methods:**

Fifty comprehensive clinical case scenarios including patient age, presenting complaint, history of the presenting complaint, medical history, allergies, intra- and extraoral findings, lesion description, and any additional information including laboratory investigations and specific clinical features were given to three oral medicine consultants, who were asked to formulate a differential diagnosis and a final diagnosis. Specific prompts for the same 50 cases were designed and input into ChatGPT and Copilot to formulate both differential and final diagnoses. The diagnostic accuracy was compared between the LLMs and oral medicine consultants.

**Results:**

ChatGPT exhibited the highest accuracy, providing the correct differential diagnoses in 37 of 50 cases (74%). There were no significant differences in the accuracy of providing the correct differential diagnoses between AI models and oral medicine consultants. ChatGPT was as accurate as consultants in making the final diagnoses, but Copilot was significantly less accurate than ChatGPT (*P*=.015) and one of the oral medicine consultants (*P*<.001) in providing the correct final diagnosis.

**Conclusions:**

ChatGPT and Copilot show promising performance for diagnosing oral medicine pathology in clinical case scenarios to assist dental practitioners. ChatGPT-4 and Copilot are still evolving, but even now, they might provide a significant advantage in the clinical setting as tools to help dental practitioners in their daily practice.

## Introduction

Creating models that accurately replicate the complexity of the human brain and thinking has been a longstanding challenge for the scientific community [1]. The term “artificial intelligence” (AI) was first coined by John McCarthy in 1956, and this evolving scientific and engineering challenge focuses on computationally understanding intelligent behavior and creating applications that demonstrate such behavior [2]. AI has also emerged as a promising avenue for enhancing the precision and efficiency of diagnosing oral lesions. The diagnosis of pathological conditions within the oral cavity has traditionally relied on visual examination, histopathological analysis, and clinical expertise [3]. However, AI algorithms have the potential to analyze various data sources, including clinical images, patient records, and radiographs, to provide valuable insights and suggestions for clinicians to facilitate the diagnosis of oral lesions [4].

ChatGPT is a recently introduced AI tool developed by OpenAI. ChatGPT is a large language model (LLM) trained with extensive data and capable of understanding and generating human-like responses accurately and consistently. ChatGPT currently operates on the GPT-4 architecture, allowing it to understand and respond to complex queries in a conversational manner [5]. ChatGPT can be used in medicine by rapidly providing appropriate answers to queries (or “prompts”), for instance, by assisting in decision-making based on up-to-date research and guidelines. There are high expectations for ChatGPT in the health sciences, including for education, research, and practice across different medical disciplines [6], and it can be embedded in various platforms.

Microsoft Copilot is another AI-driven assistant that can be accessed via a web interface or through seamless integration within the Microsoft 365 suite [5]. Leveraging LLMs and insights from Microsoft Graph, Microsoft Copilot delivers tailored support, enhancing the users’ experience across Microsoft 365 applications such as Word, Excel, and PowerPoint. Copilot offers real-time suggestions and completions based on the context of the existing request. Powered by GPT-4 Turbo, it also has access to information, enhancing its utility for up-to-date coding tasks.

ChatGPT has also been used in several areas of medicine. For example, ChatGPT provided excellent responses on basic knowledge, lifestyle advice, and treatment for cirrhosis and hepatocellular carcinoma but performed less well for diagnosis and prevention [7]. In an analysis of ChatGPT responses to 284 medical questions, the results were highly accurate but incomplete [8]. AI has also been applied to dentistry [9-11]. In endodontics, AI models have been used to explore the anatomy of the root canal system, predict the health of dental pulp stem cells, detect root fractures and periapical lesions, and predict the success of retreatment procedures [12,13]. In oral medicine, ChatGPT was used to address questions about oral potentially malignant disorders. Guidelines on oral potentially malignant disorders from scientific societies were used to create questions for input into ChatGPT, which showed moderate knowledge about oral potentially malignant disorders as assessed by specialist reviewers [6]. AI also shows promise for scheduling, patient management, managing drug interactions, predictive tasks, and even robotic endodontic surgery [14], although the cost-effectiveness, reliability, and practicality of implementation still need to be assessed before widespread adoption [11].

To the best of our knowledge, no study has examined the use of AI-powered tools (ChatGPT and Copilot) in oral medicine, especially with respect to the diagnosis of oral lesions. To address this gap, herein, we compared the accuracy of ChatGPT and Copilot with oral medicine consultants in providing differential and final diagnoses from text-based clinical case scenarios.

## Methods

### Study Design

This was a comparative analytical study conducted at the King Abdulaziz University Faculty of Dentistry in Jeddah, Saudi Arabia. The primary objective was to assess and compare the accuracy of ChatGPT and Copilot with oral medicine consultants for diagnosing oral lesions from written clinical scenarios.

### Ethical Considerations

The Research Ethics Committee-Faculty of Dentistry, King Abdulaziz University granted ethical approval (no. 209-11-23).

### Data Collection

Sixty clinical case scenarios were collected from the Oral Medicine and Oral Pathology Division of the Oral Diagnostic Sciences Department. The final diagnosis was determined on the basis of the results of laboratory investigations, radiographs, and histopathological examination. Ten cases were excluded by an external reviewer, as they were deemed to be poorly written. The remaining 50 cases included patient age, chief complaint, history of the chief complaint, medical history, allergies, intra- and extraoral findings, a description of the lesions, and any additional information, including laboratory investigations and specific clinical features. An example clinical scenario is shown in Multimedia Appendix 1. The LLMs and oral medicine consultants were not provided with the histopathological features.

**Figure 1.**
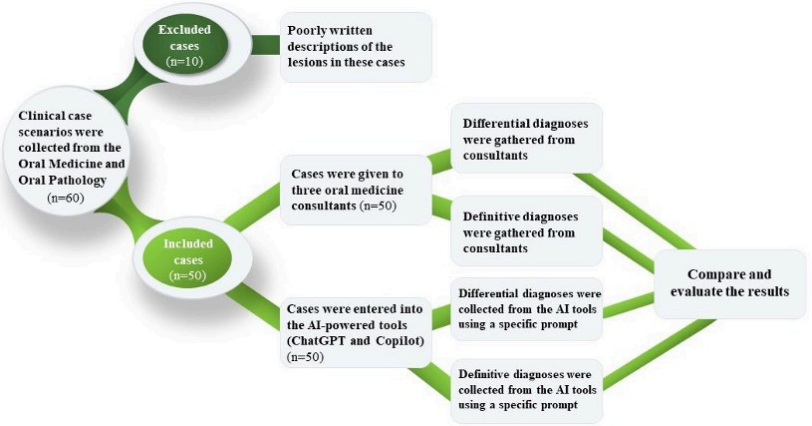
Schematic of the study design, describing the distribution of the clinical case scenarios to the oral medicine consultants and artificial intelligence (AI)-powered tools.

The cases were given to 3 oral medicine consultants (with clinical experiences of 7 years, 10 years, and 5 years for consultants 1, 2, and 3, respectively), who were asked to formulate differential and final diagnoses. Two specific prompts were designed for entry into ChatGPT and Copilot to formulate differential and final diagnoses (Figure 1): the first prompt enquired about the differential diagnoses for each clinical scenario (“As an oral medicine consultant, what is your differential diagnoses of the case?”), and the second enquired about the final diagnosis (“What is your final diagnosis based on the provided information?”).

Responses were reviewed and evaluated independently by two reviewers who specialized in oral pathology and medicine and who were involved in case selection. Any discrepancies were resolved by a third reviewer. Each response was assessed for accuracy and assigned a score based on the following criteria: the differential diagnoses responses by ChatGPT, Copilot, and consultants were assigned a score of 2 (correctly identified all the listed differential diagnoses), 1 (correctly identified one listed differential diagnosis), or 0 (wrongly identified all the listed differential diagnoses). For the final diagnosis, responses were categorized as 1 (correct) or 0 (incorrect).

### Statical Analysis

The performances of ChatGPT, Copilot, and the oral medicine consultants in providing differential and final diagnoses for oral lesions in the clinical scenarios are presented as frequency tables. The *χ*^2^ or Fisher exact test was used to compare the performance distributions between the AI tools and consultants. A *P* value of .05 was considered significant. All statistical analyses were performed using IBM SPSS Statistics version 29.0.0 (IBM Statistics).

## Results

### Comparison of Differential Diagnoses Between AI Tools and Oral Medicine Consultants

ChatGPT exhibited the highest accuracy, correctly diagnosing 74% (37/50) of the cases, partially diagnosing 24% (12/50) of the cases correctly, and making completely incorrect diagnoses in only 2% (1/50) of the cases. In contrast, Copilot provided all correct differential diagnoses for 60% (30/50) of the cases, only one correct diagnosis in 34% (17/50) of the cases, and all wrong diagnoses in 6% (3/50) of the cases. There was no significant difference in the accuracy between the two models (*P*=.32).

In comparison to these AI models, oral medicine consultant 1 correctly diagnosed 60% (30/50), partially diagnosed 34% (17/50), and incorrectly diagnosed 6% (3/50) of the cases (*P*=.32 vs ChatGPT and *P*≥.99 vs Copilot). Oral medicine consultant 2 correctly diagnosed 72% (36/50) of the cases, partially diagnosed 22% (11/50) of the cases, and incorrectly diagnosed 6% (3/50) of the cases (*P*=.75 vs ChatGPT and *P*=.41 vs Copilot). Lastly, oral medicine consultant 3 accurately diagnosed 54% (27/50) of the cases, partially diagnosed 38% (19/50) of the cases, and incorrectly diagnosed 8% (4/50) of the cases (*P*=.10 vs ChatGPT and *P*=.82 vs Copilot). The AI models had similar accuracy in providing the differential diagnoses as oral medicine consultants, as shown in Table 1.

**Table 1.** Comparison of accuracy of artificial intelligence (AI) versus oral medicine consultants for differential diagnoses.

AI model or consultant	Differential diagnosis, n (%)				
	All wrong	One correct	All correct	*P* value^a^	*P* value^b^
Oral medicine consultant 1	3 (6)	17 (34)	30 (60)	.31	≥.99
Oral medicine consultant 2	3 (6)	11 (22)	36 (72)	.74	.42
Oral medicine consultant 3	4 (8)	19 (38)	27 (54)	.11	.82
ChatGPT	1 (2)	12 (24)	37 (74)	–^c^	.32
Copilot	3 (6)	17 (34)	30 (60)	.32	–

a *P* values in comparison to ChatGPT.

b *P* values in comparison to Copilot.

c "–”: not applicable.

### Comparison of Final Diagnoses Between AI Tools and Oral Medicine Consultants

With respect to the definitive diagnoses, ChatGPT again showed the highest accuracy: 70% (35/50) correct diagnoses and 30% (15/50) incorrect diagnoses. Copilot performed less well, providing 46% (23/50) correct diagnoses and 40% (27/50) incorrect diagnoses.

Oral medicine consultant 1 correctly diagnosed 66% (33/50) of the cases and incorrectly diagnosed 34% (17/50) of the cases (*P*=.66 vs ChatGPT and *P*=.04 vs Copilot). Oral medicine consultant 2 had the highest diagnostic accuracy, diagnosing 80% (40/50) of the cases correctly and 20% (10/50) incorrectly (*P*=.25 vs ChatGPT and *P*<.001 vs Copilot). Oral medicine consultant 3 correctly diagnosed 64% (32/50) of the cases and incorrectly diagnosed 36% (18/50) of the cases (*P*=.52 vs ChatGPT and *P*=.07 vs Copilot); the data are shown in Table 2.

**Table 2.** Comparison of accuracy of artificial intelligence (AI) versus oral medicine consultants for final diagnoses.

AI model or consultant	Final diagnosis, n (%)			
	Wrong	Correct	*P* value^a^	*P* value^b^
Oral medicine consultant 1	17 (34)	33 (66)	.67	.04
Oral medicine consultant 2	10 (20)	40 (80)	.25	<.001
Oral medicine consultant 3	18 (36)	32 (64)	.52	.07
ChatGPT	15 (30)	35 (70)	–^c^	.02
Copilot	27 (54)	23 (46)	.02	–

a *P* values in comparison to ChatGPT.

b *P* values in comparison to Copilot.

c "–”: not applicable.

## Discussion

In this study, we compared the diagnostic accuracy of AI language models (ChatGPT-4 and Copilot) with three oral medicine consultants in providing differential and final diagnoses for oral lesions from text-based clinical scenarios. We found that the diagnostic accuracy of the LLMs and oral medicine consultants for providing accurate differential diagnoses was similar. However, Copilot was significantly less accurate than ChatGPT (*P*=.015) and one of the oral medicine consultants (*P*<.001) in providing the correct final diagnoses. Our results suggest that advanced language models, especially ChatGPT, can provide comparable diagnostic insights to human experts in the context of oral lesion diagnosis. ChatGPT-4 and Copilot are still evolving, but even now, they might provide a significant advantage in the clinical setting as tools to help dental practitioners in their daily practice. Copilot may have underperformed in making the final diagnoses compared to ChatGPT and consultants due to differences in training, dataset variations, and algorithmic constraints. ChatGPT is exposed to a broader range of medical and dental literature, whereas Copilot is optimized for general productivity, affecting its diagnostic precision. Additionally, Copilot’s customization for enterprise applications may limit its ability to provide accurate clinical diagnoses [15].

Our findings are consistent with those obtained by Altamimi et al [16], who concluded that AI tools can be useful in clinical settings to provide diagnoses for certain conditions. Friederichs et al [17] evaluated the performance of ChatGPT using 400 multiple-choice questions from the progress test administered in German-speaking countries, reporting that ChatGPT surpassed most first- to third-year medical students by correctly answering two-thirds of the multiple-choice questions, with proficiency equivalent to the level required for the German state licensing examination in Progress Test Medicine. Several studies have reported similar accuracy and efficacy of ChatGPT. A recent study from India demonstrated that ChatGPT was a reliable tool for addressing complex problems that involved higher-level cognitive skills such as interpretation, analysis, evaluation, and evidence-based opinion or prediction, correctly answering 100 complex questions in pathology [18]. Das et al [19] reported that ChatGPT could be considered a tool for answering direct inquiries regarding microbiology, showing 80% accuracy in its responses. Furthermore, Johnson et al [8] found that ChatGPT consistently provided accurate and comprehensive responses to a variety of questions in the medical field.

Copilot showed promising performance in providing differential diagnoses compared with oral medicine consultants, albeit with higher rates of all wrong differential diagnoses. Kaftan et al [20] recently examined the accuracy of AI-powered tools for interpreting biochemical data, reporting the highest accuracy for Copilot compared with ChatGPT-3.5 and Gemini. However, ChatGPT-3.5 had fewer capabilities than ChatGPT-4, which we used here. While Copilot is based on GPT-4, as noted above, its outputs differ due to Microsoft-specific customizations, including specialized training for productivity tasks, integration with enterprise tools, and compliance filters, perhaps explaining the difference in results between the two LLMs [20]. Tepe and Emekli [21] similarly observed significant variability between LLMs for answering prompts related to breast imaging. ChatGPT-4 showed high accuracy in responding to these questions, outperforming Gemini and Copilot. Moreover, AI-powered tools tended to give more differential diagnoses for each clinical scenario, regardless of whether the answers were all correct or not, with only two answers needed for analysis. Accordingly, expert judgment, knowledge, and experience are required to evaluate these answers to construct specific differential diagnoses for each case.

Diniz-Freitas et al [6] reported that integrating ChatGPT into oral medicine could significantly accelerate decision-making for patient diagnosis, treatment, and care. We found that oral medicine consultants outperformed Copilot with respect to the final diagnosis. However, one of the oral medicine consultants outperformed Copilot in providing an accurate final diagnosis, and this was the consultant with the most experience. Clinicians accumulate subject-specific knowledge and experience. Consequently, AI tools like ChatGPT, when paired with health care practitioners’ expertise, could yield even more dependable and efficient outcomes for patients requiring oral medicine treatment [6].

AI tools obtain their datasets from different sources and have different training, which influences their applications and affects their performance. Training AI tools with medical or dental datasets reviewed by specialists might be expected to improve results and transform diagnostic health care services. The clinician’s experience, which is influenced by solid knowledge and experience and unaffected by dataset variability, plays a major role in their superiority over AI tools [22].

As the training and refinement of filtered datasets improve AI tools, LLMs are expected to be integrated into clinical workflows, especially in areas without access to specialized consultants in the field. During the implementation of such technologies, ethical concerns should be considered and governed. The privacy and safety of patient data are major concerns in the use of AI in health care, requiring adherence to regulations like the HIPAA (Health Insurance Portability and Accountability Act) to prevent unauthorized access [23,24]. There are also medico-legal concerns, as AI-related errors could lead to liability issues, necessitating clear regulatory frameworks. Ethically, AI should serve as an assistive tool rather than a replacement for clinical expertise to maintain fairness and reliability. Clinician reliance on AI must be balanced to ensure that decision-making remains informed by human judgment, supported by proper training.

This study has some limitations. It was a pilot study that focused solely on evaluating the application of AI-powered tools in diagnosing text-based clinical scenarios specific to oral medicine. Therefore, the findings and conclusions may not be applicable or generalizable to other subjects or domains. Depending on text-based clinical scenarios makes it more difficult to provide both differential and definitive diagnoses. Using clinical images and histopathological findings greatly improves the accuracy of diagnostics, which were not provided in this study. Moreover, we only studied a limited number of cases (50 questions), and 10 cases were excluded by an external reviewer, which may have introduced bias. The formulation of the input “prompts” when interacting with language models can greatly impact the quality and nature of the generated responses. Consequently, further studies are needed to examine the optimal prompts that provide the best and most accurate responses. Moreover, it remains uncertain whether LLMs consistently produce identical or similar responses to the same query at different times. In this study, each question was submitted only once to the AI-powered tools, which may have limited the assessment of response consistency. Additional studies are needed to overcome these limitations and explore the real-world potential of using AI-powered tools in oral medicine.

In conclusion, LLMs such as ChatGPT and Copilot showed promising performance in making diagnoses in oral medicine clinical case scenarios. ChatGPT-4 and Copilot are still evolving, but even now might provide a significant advantage in the clinical setting as tools to help dental practitioners in their daily practice. Such technologies could particularly benefit dentists in rural areas or areas with no access to oral medicine consultants, who—provided the technology is further validated—could collect medical histories, perform extra- and intraoral examinations, and provide these data to LLMs systems to provide a set of relevant differential diagnoses to help with decision-making regarding further testing, referral, or simple management.

## Supplementary material

10.2196/70566 Multimedia Appendix 1 Example clinical scenario.

## ABBREVIATIONS

AI: artificial intelligence
HIPAA: Health Insurance Portability and Accountability Act
LLM: large language model
