# Supplementary material for: The Diagnostic Performance of Large Language Models and Oral Medicine Consultants for Identifying Oral Lesions in Text-Based Clinical Scenarios: Prospective Comparative Study
Source: JMIR AI. 2025 Apr 24;4:e70566. doi: 10.2196/70566 (PMC12223689; doi:10.2196/70566)
Supplement: Multimedia Appendix 1 [file ai-v4-e70566-s001.docx]

**Multimedia Appendix 1**

***Example clinical scenario***

A 45-year-old Indian female presented to the dental clinic with a painless mass in the soft palate. The mass was noted 2 months ago and has been gradually increasing in size. She denies any dysphagia or oral bleeding. The patient is healthy. Her mother and father are diabetic. She visits the dentist regularly every 6 months. She is a single student. She does not smoke. Lesion Description: Around 2 cm solitary dome shape pink mass that is presented in the left side soft palate, it is crossing the midline. Relatively it has ill-defined border, and it is extended posteriorly from the uvula up to the hard-soft palate anteriorly. it has smooth with capillary shown surface.
